# Editorial: Extra-Oral Taste Receptors: Function, Disease and Evolution

**DOI:** 10.3389/fphys.2020.607134

**Published:** 2020-10-30

**Authors:** Ronghua ZhuGe, Eugeni Roura, Maik Behrens

**Affiliations:** ^1^Department of Microbiology and Physiological Systems, University of Massachusetts Medical School, Worcester, MA, United States; ^2^Centre for Nutrition and Food Sciences, Queensland Alliance for Agriculture and Food Innovation, The University of Queensland, St. Lucia, QLD, Australia; ^3^Leibniz-Institute for Food Systems Biology at the Technical University of Munich, Freising, Germany

**Keywords:** chemosensing, taste receptors, TAS2R, sweet taste receptors, extraoral, disease

Taste is one of the five “classic” senses and initiates in taste receptor cells in the oral cavity of humans and other mammals. However, taste receptors and the molecules responsible for tastant detection are also widely expressed throughout the body, and they are involved in a wide variety of functions in tissues and organs outside the mouth. In the gastrointestinal tract (GIT), sweet taste receptors (i.e., TAS1R2+TAS1R3 heterodimer) contribute to glucose sensing and energy balancing (Jang et al., 2007; Margolskee et al., 2007); umami and other amino acids receptors (i.e., TAS1R1+TAS1R3, mGluR1, mGluR4, CaSR, GPRC6A, GPR92) reflect on protein-related nutrients (Haid et al., 2012; San Gabriel and Uneyama, 2013; Steensels and Depoortere, 2018; Modvig et al., 2019; Roura et al., 2019); Bitter taste receptors (TAS2Rs) have been involved in GIT motility, hunger/satiety hormone secretion and innate immune responses to parasite infection (Wu et al., 2002; Glendinning et al., 2008; Janssen et al., 2011; Howitt et al., 2016; Serrano et al., 2016; von Moltke et al., 2016; Kok et al., 2018). In addition, TAS1Rs are found in the hypothalamus, the main organ involved in the control of food intake, responding to the nutritional status in mice (Ren et al., 2019). In the respiratory system, TAS2R activation increases airway ciliary beat frequency (Shah et al., 2009), but paradoxically relaxes airway smooth muscle (Deshpande et al., 2010; Zhang et al., 2013). In the genitourinary system, TAS2Rs participate in spermatogenesis (Li and Zhou, 2012) and mediate a reflex loop in the urethra, leading to bladder contraction (Deckmann et al., 2014). In the cardiovascular system, TAS1Rs and TAS2Rs were found in cardiac myocytes, the latter being upregulated following starvation (Foster et al., 2013). Intriguingly, the expression of TAS1Rs and TAS2Rs is altered under several pathological conditions, and their polymorphisms have been linked to several human disorders (Lee et al., 2012; Orsmark-Pietras et al., 2013).

This Research Topic provides a timely overview of the latest insights into the extra-oral taste receptors in health and disease. Nayak et al. provide a comprehensive and systematic review of the expression and function of TAS2Rs in different airway and lung cells. They discussed the diverse effects of TAS2Rs in mitigating various pathological features of asthma and highlighted specific opportunities for developing selective agonists for distinct TAS2R subtypes in the treatment of asthma. Grassin-Delyle et al. presented new evidence on the expression of TAS2Rs in human macrophages and demonstrated that the agonists of TAS2R3, 4, 5, 9, 10, 14, 30, 39, and 40 inhibit cytokine production induced by lipopolysaccharide. Their results expand the cell type expressing TAS2Rs in the lung and substantiate the idea that TAS2Rs may constitute new drug targets in inflammatory obstructive lung disease. Luo et al. evaluated the potential value of screening TAS2R agonists that relax smooth muscle based on the bitter flavors of Traditional Chinese medicines (TCM). The authors applied bioinformatics mining to TCM databases and discovered many bitter tastants from TCM, which can activate TAS2Rs, as new smooth muscle relaxants. Bloxham et al. highlighted current research on bitter taste receptors and their signaling cascade in the heart. They stressed the need further to study these receptors' functions in the heart and predicted that TAS2Rs may involve in unappreciated cardiac physiology.

Fat or fatty acids have been recognized as the triggers of a potential sixth taste in animals and humans. Similar to the TAS1R and TAS2R families, the receptors for fatty acids, e.g., CD36, GPR40 (FFAR1), GPR41 (FFAR3), GPR43 (FFAR2), GPR84, and GPR120 (FFAR4), are also expressed in many cell types throughout the body. In the GIT and hypothalamic nucleus, fatty acids act on these receptors to critically regulate energy balance by changing ingestive behavior, energy storage, and utilization (Blouet and Schwartz, 2010; Cvijanovic et al., 2016; Liu et al., 2016). Significantly, these receptors' sensitivity to fatty acids in obese individuals is lower than that in lean individuals. This may account for excess fat consumption in obese individuals and contribute to other diseases such as type 2 diabetes (Stewart et al., 2011; Ichimura et al., 2012; Precone et al., 2019). Le Foll updated the current understanding of hypothalamic fatty acids and ketone bodies sensing (i.e., FAT/CD36 and GPCRs) and enzymatic activity (e.g., LPL) in regulating food intake. In particular, the author highlights that the expression of FA sensors/transporters in neurons, astrocytes, and tanycytes mediates the regulatory function. However, other aspects linked to obesity and/or Type 2 diabetes, such as the impact of inflammation on FA and ketone bodies sensing remain to be further investigated.

We believe this Research Topic provides an exciting overview of the extraoral taste receptors. Since these receptors mediate the critical functions of extraoral tissues and contribute to different diseases, we hope that this Topic will foster the discovery of new pharmaceutical therapeutics targeting these taste receptors for various human diseases and disorders.

## Author Contributions

RZ, ER, and MB co-wrote and approved this Editorial. All authors contributed to the article and approved the submitted version.

## Conflict of Interest

The authors declare that the research was conducted in the absence of any commercial or financial relationships that could be construed as a potential conflict of interest.
